# Color vision improves foraging success and accelerates reproduction in a jumping spider

**DOI:** 10.1093/beheco/arag110

**Published:** 2026-09-15

**Authors:** Erin C Powell, Kayla Stalcup, Lisa A Taylor

**Affiliations:** Florida State Collection of Arthropods, Florida Department of Agriculture and Consumer Services, Division of Plant Industry, 1911 SW 34th St., Gainesville, FL 32608, United States; Entomology and Nematology Department, University of Florida, 1881 Natural Area Drive, Gainesville, FL 32611, United States; Entomology and Nematology Department, University of Florida, 1881 Natural Area Drive, Gainesville, FL 32611, United States; Entomology and Nematology Department, University of Florida, 1881 Natural Area Drive, Gainesville, FL 32611, United States

**Keywords:** Salticidae, aposematism, predator–prey interactions, reproductive fitness, learning, visual ecology, decision-making

## Abstract

Color vision is thought to convey fitness advantages during foraging, but empirical tests of this idea are exceedingly rare. We manipulated prey color cues in tests with the trichromatic jumping spider *Habronattus pyrrithrix* to assess whether their ability to use their color vision affected foraging success and reproduction. Mated adult female spiders were first trained to associate the color red with experimentally manipulated unpalatable (bitter) prey and then tested to confirm that they learned this association (independent of brightness cues). They were then divided into 2 treatment groups. Both groups were given a similar foraging task where they foraged among arrays of palatable and unpalatable prey. One group was given red color cues to make these choices (as in training, red signaled unpalatability, independent of brightness), while the other group did not have red color cues to make these choices. Spiders with access to color cues targeted the palatable prey more accurately, consumed more palatable prey during their trial period, and tended to direct fewer wasted attacks toward unpalatable prey. Over a 2-mo period, females with consistent access to color cues laid eggs earlier and tended to be heavier, but the total number of egg sacs, eggs, and hatchlings produced was unaffected. This study is among the first to directly link color vision with foraging success and fitness in any animal. Future work should track total lifetime reproductive output, test benefits of color vision across ecological contexts, and explore fitness consequences across the salticid phylogeny.

## Teaser

Seeing red helps spiders hunt smarter. We trained jumping spiders to associate red with bitter prey and gave them repeated foraging challenges to ask how the ability to see red may have improved their success. Spiders using color vision consumed more prey, made fewer mistakes, and laid eggs sooner. But, after two months, it didn't affect how many offspring they produced, suggesting that the full fitness benefits of color vision may emerge over longer timescales.

## Introduction

Color vision has evolved repeatedly across animals, likely driven by diverse selective pressures (Kelber et al. 2003; Kelber and Osorio 2010; van der Kooi et al. 2021). For example, the ability to see and discriminate color likely plays critical roles in the contexts of signaling mate quality (Andersson 1994; von Schantz et al. 1999; Hill 2006), locating specific foods (Lunau and Maier 1995; van der Kooi et al. 2019; Taylor et al. 2022), avoiding unprofitable prey (Gamberale-Stille and Tullberg 1999), achieving crypsis through background matching (Merilaita and Stevens 2011), detecting predators (Pessoa et al. 2014), locating nests (Somanathan et al. 2008), and choosing oviposition sites (Ogawa et al. 2013). Despite this widespread importance and decades of research on the evolution and functions of color vision, manipulative experiments that enable us to quantify the fitness benefits of color vision remain elusive. While there is a clear call for such empirical studies (Surridge et al. 2003; Bradley and Lawler 2011), they are still exceedingly rare in any taxa (Fedigan et al. 2014; Caves et al. 2025).

In the absence of manipulative experiments, other approaches have been used to provide some insights into fitness benefits of color vision in the context of foraging. For example, in some primate species, individuals within the same population and even the same sex differ in their color vision (Surridge et al. 2003). Some are dichromatic with limited color discrimination ability, while others are trichromatic and able to distinguish a broader range of colors, most notably red from green. Because these individuals live in the same environment and belong to the same species, it is possible to directly test whether trichromatic individuals gain fitness advantages over dichromatic individuals. In capuchin monkeys, such approaches have shown that trichromatic individuals consume more reddish fruit than their dichromatic group mates (Melin et al. 2017). Yet studies gathering long-term fitness data on a sister species of capuchin monkey found no evidence of higher fertility rates, number of offspring, or maternal survival over a 26-yr field study (Fedigan et al. 2014). While trichromatic individuals may be better at finding nutrient-rich foods such as ripe figs, there is no actual net increase in feeding rate, suggesting that dichromats may be compensating in other ways (eg using olfaction) (Melin et al. 2009). Additional work shows that dichromats might sometimes have visual advantages over trichromats; under low-light conditions, dichromats are more efficient than trichromats at breaking color camouflage (ie detecting surface-dwelling insects whose color is closely matched to that of their background) (Melin et al. 2007). In marmosets, dichromatic individuals outperform trichromats while foraging in the shade, but not the sun (Caine et al. 2010). A recent longitudinal study on spider monkeys found no effect of color vision (trichromatic vs. dichromatic) on reproductive output over an 18-yr period (Rettke 2023). Taken together, these findings suggest that both dichromacy and trichromacy may provide advantages in different contexts, perhaps allowing both to persist over evolutionary time, but this work remains in its infancy (Hiwatashi et al. 2010; Fedigan et al. 2014). Outside of this body of work on primates, similar, but limited, work in fish has found that mutant zebrafish with reduced UV cones (for short-wavelength vision) have diminished foraging performance compared with wild-type individuals (Flamarique 2016). While it seems intuitively obvious that color vision should improve foraging, evidence for nuanced tradeoffs outlined in the work above, should caution us against assuming such benefits exist without directly testing them.

One way that color vision, and particularly long-wavelength color vision, might aid foraging predators is by helping them avoid aposematic prey (ie unprofitable or dangerous prey that advertise their defenses with long-wavelength colors like oranges and reds, reviewed in Ruxton et al. 2004; Mappes et al. 2005). Attacks on such prey can carry high costs, including wasted handling time, toxin exposure, and other forms of injury, as documented in birds consuming monarch butterflies (Brower 1969) or lady beetles (Marples et al. 1989) or in toads that ingest bombardier beetles (Sugiura and Sato 2018). For visual predators, the ability to perceive and discriminate long-wavelength colors should help them recognize colorful warning displays and avoid them, either through innate or learned color aversions (Stevens and Ruxton 2012). By contrast, predators with limited color vision must rely more heavily on other cues (Raška et al. 2018; Halpin et al. 2020; Humbel et al. 2023; Stuckert and Summers 2023) potentially increasing the likelihood of error. As a result, predators with color vision may gain fitness benefits that come from avoiding defended prey and increasing their foraging on profitable prey.

Jumping spiders (family Salticidae) are a powerful system to test the fitness advantages of color vision in this foraging context. As the most diverse spider family with 6,950 species worldwide (World Spider Catalog 2026), these tiny voracious predators are unique for their impressive visual acuity in their forward-facing principal eyes (Williams and McIntyre 1980) and their repeated independent evolution of true color vision (ie the capability to discriminate among objects based on difference in color rather than brightness) (Outomuro et al. 2019). Several species are known to use colorful signals in the contexts of mate choice (Taylor and McGraw 2013; Yan et al. 2025) and male–male interactions (Lim and Li 2004; Cross et al. 2020). They also attend to long-wavelength color cues (eg red) to locate specialized prey such as blood-fed mosquitos (Taylor et al. 2022) and to avoid unpalatable aposematic prey (Taylor et al. 2014, 2016; Powell et al. 2019; Vickers and Taylor 2020).

The North American genus *Habronattus* F. O. Pickard-Cambridge has a particularly unique visual system unlike any other jumping spider clade. They have intraretinal filters that enable trichromatic vision, improving their ability to see and discriminate long-wavelength colors, such as red (Zurek et al. 2015). This visual system is likely responsible for their ability to learn generalized color biases following positive and negative experiences with colorful prey (Taylor et al. 2014, 2016; Powell et al. 2019). Prior work on *Habronattus pyrrithrix* (Chamberlin) has streamlined species-specific methods for experimentally manipulating both color and palatability in prey choice and learning experiments (Taylor et al. 2014, 2016; Winsor et al. 2020). Moreover, the short life spans of these jumping spiders (likely about 1 yr from hatching to senescence in the field, pers. obs.), relative to other well-studied animals, provide a unique opportunity to capture measures of fitness that encompass a large portion of the animal's natural reproductive life.

Our goal in the present study was to test the hypothesis that color vision (ie the ability to use color cues) enhances foraging success and fitness in *H. pyrrithrix*. Because we cannot manipulate the visual system of these spiders directly, we designed a manipulative experiment where we instead manipulated the spiders' ability to use color cues to navigate prey options and to use these color cues (or not) to avoid unpalatable prey and find palatable prey. This study consisted of 3 phases. In Phase 1 (training), we fed mated adult female spiders a training diet to teach them to associate the color red with unpalatable prey. At the end of this phase, we confirmed that the spiders did indeed avoid red prey (independent of brightness), consistent with their training. In Phase 2, we randomly assigned these spiders to 1 of 2 treatment groups. Both groups were given a similar foraging task where they had to forage among several prey that were either palatable or unpalatable. However, 1 group was given red color cues to make these choices (as in training, red signaled unpalatability, independent of brightness), while the other group did not have red color cues available to make these choices. This phase of the experiment allowed us to test the hypothesis that the ability to use color cues improves foraging success. In Phase 3, we assessed longer-term consequences of foraging success by measuring whether differences in the ability to use color cues over 2 mo of experimental manipulation affected how soon spiders laid their first egg sac, the total number of eggs laid, and the number of offspring produced.

## Materials and methods

Prior to data collection, we preregistered our hypothesis, methods, and a priori plans for data analysis with the Center for Open Science. Preregistered analyses that directly test our hypotheses are hereafter referred to as “confirmatory analyses,” while all other analyses are considered “exploratory.” Our preregistration document is freely available to readers at the following link: https://osf.io/qswm5/. The goal of preregistration is to clearly articulate a priori hypotheses and predictions and planned analyses before collecting data; this increases the credibility of the work by clearly differentiating between a priori hypothesis testing and post hoc exploration of data (Ihle et al. 2017; Nosek et al. 2018) and is specifically encouraged by the journal *Behavioral Ecology* (Simmons 2017).

### Ethical note

No formal ethical approval was required for work with the invertebrates in our study, but we followed best practices to minimize stress to the animals. Our lab practices align with the recommendations for the ethical treatment of research animals in our field (ASAB Ethical Committee/ABS Animal Care Committee 2024).

### Spider collection

Immature female *H. pyrrithrix* jumping spiders were collected from Queen Creek, Arizona, USA in October 2020. They were fed half their own body size in crickets (*Gryllodes sigillatus* [Walker]) 3 times weekly and housed individually in clear plastic boxes (10.16 × 10.16 × 12.86 cm) with a ∼10-cm-long artificial green plant affixed for enrichment (Carducci and Jakob 2000). These boxes are opaque on one side to prevent spiders from seeing or interacting with one another. The laboratory was supplemented with full-spectrum lighting (SoLux PAR38 3500K 90W) and natural light from a large adjacent window because color vision appears to be light-limited in this group, where long-wavelength colors are best perceived under natural sunlight (Taylor and McGraw 2013; Zurek et al. 2015).

### Mating

Once the immature females reached maturity in the lab (Fig. 1a), they were mated 9 to 12 d later (which is within the time window that unpublished data suggest they are most receptive). We chose to use mated females for this experiment for several reasons. First, mated females are particularly voracious and motivated to feed (more so than juveniles or males), ensuring that they would reliably eat their training prey and reliably participate in their foraging tests. Moreover, using mated females allowed us to capture various meaningful fitness-related metrics (eg time to lay eggs, number of eggs and spiderlings produced; see details below). Finally, if we had started our experimental manipulation with immature females that were then allowed to mate and lay eggs, we would have expected those with access to color cues to forage more successfully and therefore reach maturity faster; in this case, we would not have been able to control for the total time that females were exposed to our experimental manipulations before the onset of egg laying.

**Figure 1:**
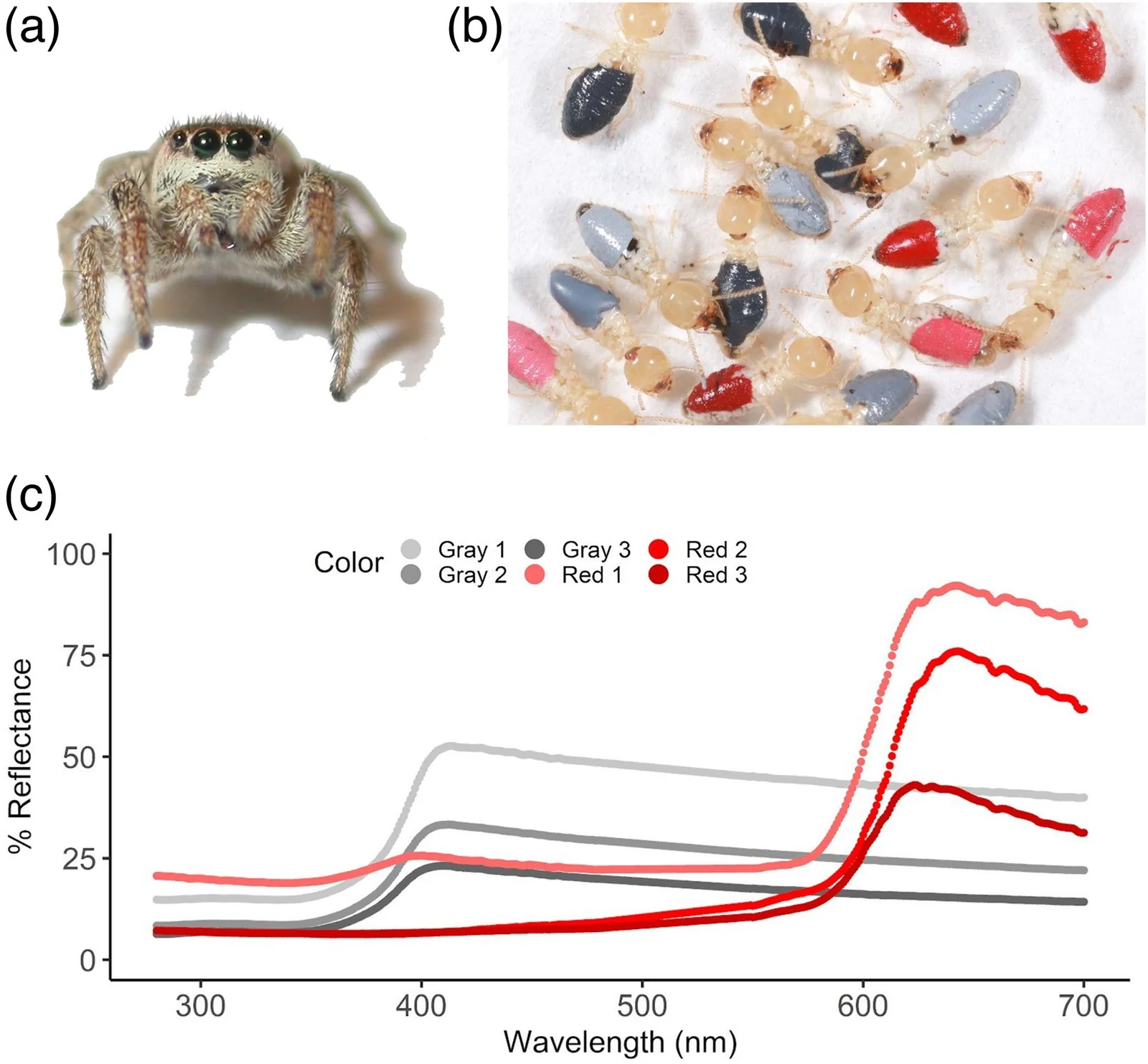
Color manipulation methods for termite prey (*R. flavipes)* presented to jumping spiders (*H. pyrrithrix*) during our experiments. (a) Adult female *H. pyrrithrix* showing the 2 enlarged, forward-facing principal eyes that have trichromatic color vision. (b) Painted termites showing varying brightness levels of red and gray. (c) Spectral properties of red and gray paint mixtures used to paint termites. Spectral curves represent the mean values of 10 measurements of each paint sample. For measurement, paint samples were applied to matte black paper, allowed to dry fully, and then measured using a UV-vis spectrophotometer (USB 2000 with PX-2 pulsed xenon light source, Ocean Optics, Dunedin, FL) with a consistent distance (3 mm) between the probe and sample for each measurement. We removed artifacts between the 480 and 550 nm range caused by oversaturation from the light source using the function CLRspike designed for this purpose in the CLR v1.05 software (Montgomerie 2008). Mean reflectance (%) values were R1 = 37.73; G1 = 38.02; R2 = 22.95; G2 = 22.64; R3 = 15.48; G3 = 15.51. Note that the colors used for (c) are for illustrative purposes only and are similar but not spectrally matched to the paint colors used in the study.

Each female was mated with a unique individual male, randomly selected from a cohort of males collected from the same field site. If unique males were not available for every female (due to natural mortality of males over the span of the study), we randomly selected males to be used again (as males will readily mate repeatedly). Females of *Habronattus* appear to usually only mate once (Rivera et al. 2021; pers. obs.). Males were added to the female's home box: a clear plastic box (10.16 × 10.16 × 12.86 cm) with a ∼10-cm-long artificial green plant. We observed mating pairs for 8 h using video cameras to ensure mating had taken place. If we did not observe mating during this timeframe, the female was paired with a new randomly selected male every day until she had mated.

Fifty-four adult females mated successfully (this included all spiders that were available to us from our field-collection trip), but 5 died in the lab prior to the first data collection point. Therefore, data from 49 females were included in our analyses.

### Manipulation of prey color

Termites used as the prey in our experiments (*Reticulitermes flavipes* [Kollar]) were field-collected in Gainesville, Florida, United States, and painted using Testors enamel paints (The Testor Corporation, Rockford, Illinois; flat red: 1150_RM11501_0611; flat black: 1149_RM11491_0611; flat white: 1168_RM11681_0611) using methods modified from Powell et al. (2019) (Fig. 1b). We painted the termites at least 10 min before presenting them to the spiders to ensure the paint was fully dry. We created 3 pairs of red and gray paint mixtures (R1 and G1, R2 and G2, R3 and G3); members of each pair were matched in mean brightness (within the spiders' visible range, 280 to 700 nm; see Peaslee and Wilson 1989; Li et al. 2008) following methods described by Montgomerie (2006) for quantifying mean brightness, independent of the visual system of the viewer (Fig. 1). We developed these mixtures by adding white or black paint to the red paint and white to the black paint. While recent work has provided insight into the visual system of *H. pyrrithrix* (Zurek et al. 2015), our current knowledge of brightness perception remains limited (eg it is still unclear which photoreceptors mediate brightness perception). For this reason, our goal was not to create precisely brightness-matched pairs as the spiders perceive them, as we cannot yet do this with certainty (see visual modeling section below). Instead, our goal was to create gray and red termites of varying brightness levels. With this design, we ensured that spiders would be most reliably able to avoid the unpalatable prey by relying on red color cues (ie chromatic cues) while making brightness cues (ie achromatic cues) alone relatively unreliable. We used a UV-vis spectrophotometer (USB 2000 with PX-2 pulsed xenon light source, Ocean Optics, Dunedin, Florida) and a series of *t*-tests to confirm that our red and gray paint pairs (3 reds and 3 grays, 10 samples each) were statistically similar in mean brightness (R1 = G1, R2 = G2, R3 = G3) while the different brightness levels were statistically different in mean brightness from one another (eg R1 > R2 > R3, G1 > G2 > G3) (Table S1).

### Visual modeling

In addition to the approach above, we also used pavo v2.9.0 (Maia et al. 2019) in R 4.5.2 (R Core Team 2026) to construct the best available visual models to analyze how these color cues likely appeared in the visual system of *H*. *pyrrithrix*. In pavo, reflectance spectra were trimmed to 300 to 700 nm (the spectral sensitivity of this species; see Zurek et al. 2015), laboratory illuminance spectra were converted to photon flux, and the reflectance spectrum of the white filter paper used in our experimental setup (see details below) was used as the background. Visual models were constructed using the built-in *H*. *pyrrithrix* spectral sensitivities (from UV [short wavelength] to long wavelength), ocular transmission data, and photoreceptor densities (s:m:l) of 1:2:4 (following Zurek et al. 2015). Models were run without von Kries adaptation (because illuminance was constant in the lab setting). We ran 4 permutations of the models to account for different possible mechanisms of brightness perception, depending on which photoreceptors mediate it (ie with the achromatic channel based on the short [s], medium [m], or long [l] wavelength photoreceptor classes or all photoreceptor classes). Quantum catches were Fechner-transformed (logarithmic scale accounts for perceptual saturation at high luminance) and scaled to 100 (relative scaling was not applied). Color distances (just noticeable differences or JNDs) were calculated using receptor-noise limited models, assuming quantum noise and including the 4 achromatic channels listed above, with the Weber fraction set to 0.05 (following Zurek et al. 2015) (Tables S2, S3, S4). All visual model code and relevant data are available in Dryad (see Data Accessibility Statement).

We used these visual models to assess 3 things related to the soundness of our experimental design. First, we confirmed that the *H. pyrrithrix* visual system should easily allow these spiders to discriminate any of our red termites from any of our gray termites using chromatic (redness) differences alone (Table S4). Second, we confirmed that these visual models, at present, do not allow us to confidently control for brightness as the spiders perceive it. This is because depending on which photoreceptors are set to mediate brightness perception (short [s], medium [m], long [l] or all), we get vastly different results. For example, if the long-wavelength photoreceptor or all photoreceptors are set to mediate brightness perception, then our red paints should, on average, appear brighter to the spiders than our gray paints (Tables S2, S3). In contrast, if the short or medium wavelength photoreceptors are set to mediate brightness perception, then our gray paints should, on average, appear brighter to the spiders than our red paints (Tables S2, S3). Given this uncertainty in the models, the best approach at this stage is to simply vary the brightness levels of both red and gray in our experiment (as we have done) such that we can be confident that the most reliable signal of unpalatability is redness, rather than brightness. The third pattern that our models confirmed was that our manipulation of brightness (within both red and gray) was substantial given the *H. pyrrithrix* visual system; high JNDs between the different brightness levels of paints suggest that the spiders could easily discriminate them based on brightness alone. With this approach, we can analyze how the spiders respond to variation in both redness and brightness independently (see Statistical Analysis section for our behavioral experiment below).

### Manipulation of prey palatability

To make prey unpalatable, we applied a 3% denatonium benzoate (DB) solution (Bitrex) (Chemsavers, Bluefield, VA, USA) to termite prey following methods outlined in Winsor et al. (2020). During Phase 1 of the study (training phase), DB was applied to the red termites only. In Phases 2 and 3 of the study, it was applied to both red and gray termites (depending on treatment; see below). We know from previous studies that DB treatment does not affect termite behavior (ie movement rate) and it is not detectable to the spiders before they attack. Both naive spiders and those that had already experienced DB attacked DB and non-DB termites at equal rates, suggesting that there are no aversive volatile chemical cues that the spiders detect before making contact with the prey that might affect their choices (Winsor et al. 2020). DB is extremely bitter at even micro concentrations, and so we took care to prevent contamination while handling and applying it to the termites. To avoid DB-treated termites coming into contact with non-DB-treated termites (and making them unpalatable), we used small individual Petri dishes (1 cm tall × 3.5 cm diameter) lined with damp white filter paper to individually contain termites to ensure they could not interact. Damp filter paper in their Petri dishes prevented the termites from desiccating.

### Phase 1: training spiders to associate red with unpalatability

Immediately after an individual spider was mated, she began training to associate red prey with unpalatability following methods modified from Taylor et al. (2016) and Winsor et al. (2020). Three times weekly (Monday, Wednesday, Friday [MWF]), beginning on the first M,W, or F after the female mated, she received 2 painted termites: 1 gray untreated termite (palatable) and 1 red termite treated with DB (unpalatable) in separate 3.5 cm diameter Petri dishes. For each spider and each feeding bout, we selected 1 red and 1 gray termite randomly from the 3 brightness categories described above (R1, G1, R2, G2, R3, G3). This allowed us to train the spiders to pay attention to the color (red or gray) rather than the brightness level. The individual termite dishes were introduced to the floor of the spiders' houses during feeding. We randomized the position of red and gray termite dishes so spiders did not learn that one side or position was the palatable termite (instead paying attention to the color cue). In addition to DB-treated red termites, we complemented the red aversion training using red chemically-defended milkweed bug nymphs (*Oncopeltus fasciatus* [Dallas]) because previous work has shown that exposure to these distasteful bugs contributes to the development of a generalized aversion to red prey in *H*. *pyrrithrix* (Taylor et al. 2016). While naive *H*. *pyrrithrix* will readily attack these bugs, they immediately reject them on contact and will avoid them on future encounters (Taylor et al. 2016). We placed 3 nymphs (∼3 mm) in the spiders' home boxes on the days between termite feedings. To prevent milkweed bugs from spreading DB onto untreated termites, milkweed bugs were only present when termites were not. We kept each spider on this training regime for 4 wk (ie 12 feedings).

At the end of the 4-wk training period, we gave the spiders a foraging task to confirm that they had indeed learned the association between red coloration and unpalatability (independent of brightness). To do this, we presented spiders with an array of 6 painted termites (R1, G1, R2, G2, R3, G3) presented in a random order in individual 3.5 cm Petri dishes on the floor of their box and gave them 2 h to forage on these termites. During these tests, the spider boxes were set upon (and surrounded by) white paper to provide a consistent visual background and to prevent visual distractions. Before the tests began, spiders were given a 10-min acclimation period where they were placed in a clear plastic petri dish in the center of the array of termite dishes. We video recorded all tests using overhead cameras so that data could later be collected from videos by an observer blind to the treatment group of the spider being tested. The presence of any color in the video (that might reveal the treatment group) was obscured by watching and scoring the videos at low resolution where the color appeared as grayscale. After the data were scored, the observer referenced a key that indicated the color/brightness category for each termite.

Using this approach, we recorded which termite (color and brightness level) was attacked first, as well as which termites were attacked across the entire 2-h trial. If spiders did not attack any termites during the 2-h trial, they were retested the following day (and continuously retested up to 3 times) until they attacked at least 1 termite. During these tests, all red termites (R1, R2, R3) were treated with DB while all gray termites (G1, G2, G3) were untreated. Previous work has demonstrated that the presence of DB does not deter attacks; spiders only show an aversive response *after* the attack occurs and the spider makes physical contact with the DB (Winsor et al. 2020). So, if the spiders avoid attacking the red termites, we can conclude that this is likely a result of the spider avoiding the red coloration, rather than avoiding the DB (due to odors or other cues from the DB). The reason for continuing to have the red termites treated with DB during these tests is that we wanted the spiders to continue to learn and maintain the association between red coloration and unpalatability for Phases 2 and 3 of the study.

### Phase 2: assessing whether the ability to use color vision improves foraging success

At the start of Phase 2, we divided the spiders randomly into 2 treatment groups. Both groups were given a foraging task similar to the test at the end of Phase 1 where they had to choose between an array of 6 termites (some of which were palatable and some of which were unpalatable) again presented in a random order in individual 3.5 cm Petri dishes on the floor of their box. For 1 treatment group (“color-present group”), this test was identical to the test given at the end of Phase 1, where the spiders had red color cues available to them to help them avoid the DB-treated termites. In this group, red coloration was always paired with unpalatability (as it was in Phase 1). The other treatment group (“color-absent group”) did not have red color cues available to make these choices. In this group, the 3 DB-treated and 3 untreated termites were gray (G1, G2, or G3). As in Phase 1, if spiders did not attack any termites during the 2-h trial, they were retested the following day (and continuously retested up to 3 times) until they attacked at least 1 termite.

This phase of the experiment allowed us to test the hypothesis that the ability to use color (specifically, the ability to use the color red as a reliable signal of unpalatability) improves foraging success. If so, we expected that the color-present group would be better able to avoid the DB-treated termites and would be better able to find and consume the palatable (untreated) termites compared with the color-absent group. Here we recorded which termite was first attacked (DB or non-DB treated), the total number of non-DB–treated termites that were consumed (with consumption being defined as the spider feeding on the termite for more than 2 min) and the total number of wasted attacks (attacks and rejections directed at DB-treated termites). Here again, all data were recorded blind to treatment.

### Phase 3: assessing whether the ability to use color vision improves fitness

During Phase 3 of the study, spiders were repeatedly given the same foraging task described in Phase 2, where spiders in the color-present group had access to red color cues to help them avoid the DB-treated termites and spiders in the color-absent group did not have access to these color cues. The goal of this phase of the study was to assess the longer-term effects of the ability to use color cues on spider fitness. Spiders completed their foraging task 3 times weekly (MWF) for a 60-d period (ie 25 times for each spider). Spiders were misted 3 times weekly with water, but we did not feed them any additional food outside of the termites they captured during their foraging tasks. Due to the number of spiders that needed to complete this task on the same day in our experimental pipeline, we did not record data during each of these repeated foraging tasks; instead, our rationale here was that if the spiders in the color-present group were indeed consistently more successful in foraging on the palatable termites (as we expected), this would be evident from our metrics of fitness (see below).

Throughout the 60-d period, we monitored the spiders daily for egg sacs and recorded how long it took them to lay their first egg sac, the total number of egg sacs produced, the total number of eggs produced, and the total number of spiderlings that emerged from the egg sacs by the end of the 60-day period.

### Statistical analyses

*P*-values (α = 0.05) were used to determine significance. For our confirmatory analyses (see description of preregistration above), we used 1-tailed tests. We used this approach because it provided the most power to test our hypotheses and because our data met the 2 explicit criteria recognized by Ruxton and Neuhäuser (2010) as appropriate for the use of 1-tailed tests. Specifically, all of our a priori predictions were explicitly directional (and preregistered as such before data collection with the Center for Open Science) and a null result or a significant result opposite the expectation would result in the same conclusion (ie rejection of our hypotheses). For exploratory analyses (ie those not explicitly included in our preregistration document as confirmatory), we used 2-tailed tests. We used parametric tests except when data violated assumptions; in these cases, we used nonparametric equivalents (eg Wilcoxon signed rank and rank sum tests, as described below). All tests were run in JMP Pro 18.0.2. Figures were made using the ggplot2 package v4.0.1 (Wickham 2016) in R 4.4.2 (R Core Team 2026).

### Statistical analyses for Phase 1: training spiders to associate red with unpalatability

#### Confirmatory tests

If the spiders learned the association between red color and unpalatability, we expected them to be less likely to direct their first attack toward a red termite than a gray one during their Phase 1 foraging challenge. We used a 1-tailed binomial test to test the directional prediction that the number of first attacks directed toward red termites was lower than the number of first attacks directed toward gray termites. Moreover, we expected them to attack fewer red than gray termites in the 2-h period. We used a 1-tailed Wilcoxon signed rank test (the nonparametric equivalent to a paired *t*-test) to test the directional prediction that spiders attacked fewer red termites than gray termites over the 2-h trial.

#### Exploratory tests

In addition to our planned confirmatory tests, we also wanted to further explore the idea that the spiders avoided the red termites, regardless of the brightness level of the color. Our experimental design involved 3 brightness levels of red (R1, R2, R3) and 3 brightness levels of gray (G1, G2, G3), and this was specifically done to make redness, rather than brightness, the most reliable indicator of unpalatability. To rule out the idea that the spiders were biasing their attacks toward brighter or darker colors (independent of redness), we ran 2 tests. First, we used the brightness level (level 1, 2, 3) as a categorical predictor variable and a likelihood ratio *X*^2^ test to ask whether there were differences between the brightness categories in which termite was first attacked. We then ran a second test where the brightness level (1, 2, 3) was treated as a continuous variable and used a *t*-test to ask whether there was a bias toward lighter or darker shades (with the null hypothesis of no bias meaning that the mean brightness level did not differ from the middle value of 2).

We also qualitatively assessed pairwise comparisons to explore the expectation that the spiders should always choose the gray over the red termite, regardless of which termite is brighter. We made these comparisons across every possible R–G combination in the data (9 comparisons). There are 2 comparisons that are of specific interest. First, when we compare R1 vs. G3, we know that R1 must be either brighter than or similarly bright to G3, regardless of which photoreceptors mediate brightness perception; this is because every point on the R1 curve is higher than every point on the G3 curve meaning that there is no possible model that would make G3 appear brighter than R1 (see Fig. 1c). Likewise, when we compare R3 vs. G1, we know that G1 must be either brighter than or similarly bright to R3, regardless of which photoreceptors mediate brightness perception; this is because every point on the G1 curve is higher than (or similar to) every point on the R3 curve meaning that there is no possible model that would make R3 appear brighter than G1 (see Fig. 1c). Because subsetting the data for these analyses resulted in low sample sizes per test (as expected and described in our preregistration document), we use these tests only to ask whether the direction of these effects is consistent across color categories rather than assessing the significance of each of these comparisons alone. We designated these tests as exploratory in our preregistration because we expected that the sample sizes might be small for each comparison (which they were); it was impossible to predict the exact sample size (and therefore the power of each test) without the data in hand. Despite the low power of some of the individual tests, assessing these overall patterns in the data provides valuable insight.

### Statistical analyses for Phase 2: assessing whether the ability to use color vision improves foraging success

#### Confirmatory tests

We first tested the prediction that the spiders' ability to avoid the DB-treated termites depended on whether they could use color cues to make that choice. We used a 1-tailed 2-sample test for proportions to test the directional prediction that spiders in the color-present treatment group were more likely to avoid the DB-treated termite on their first attack (compared with those in the color-absent group). Our second prediction was that the spiders' ability to use color cues affected how efficiently they could find (and eat) the palatable termites. We used a 1-tailed Wilcoxon rank sum test to test the directional prediction that spiders in the color-present group would successfully consume more palatable (non-DB) termites in the 2-h trial compared with those in the color-absent group. Finally, we predicted that spiders in the color-present group would have directed less wasteful attacks (ie attacks on DB-treated termites that resulted in prey rejection). We define “prey rejection” as the spider dropping the prey within 2 min of an attack (following methods in Winsor et al. 2020). We used a 1-tailed Wilcoxon rank sum test to test the directional prediction that those in color-absent group had more total wasted attacks during the 2-h trial (compared with those in the color-present group).

### Statistical analyses for Phase 3

#### Confirmatory tests

To assess whether access to color cues improves spider fitness, we used 1-tailed Wilcoxon rank sum tests to test the directional predictions that spiders in the color-present group (1) produced their first egg sac in a shorter amount of time (fewer days), (2) produced more egg sacs, (3) produced more total eggs, and (4) had more total spiderlings emerge by the end of the 60-d period compared with the color-absent group.

#### Exploratory tests

We used a 2-tailed *t*-test to ask whether spiders in the color-present group were heavier at the end of the experiment (reflecting increased foraging success and possibly indicating that they would soon lay another egg sac) compared with the spiders in the color-absent group.

## Results

### Phase 1: training spiders to associate red with unpalatability

#### Confirmatory tests

As expected, after their initial training period where they learned to associate red with unpalatability, fewer spiders directed their first attacks toward red termites compared with gray (15 attacked red, 34 attacked gray; 1-tailed binomial test, *P* = 0.0047, Fig. 2a). Also as expected, spiders attacked the red termites fewer times over the entire 2-h trial compared with gray (1-tailed Wilcoxon signed rank, *S* = 256.50, *P* = 0.0035, Fig. 2b).

**Figure 2:**
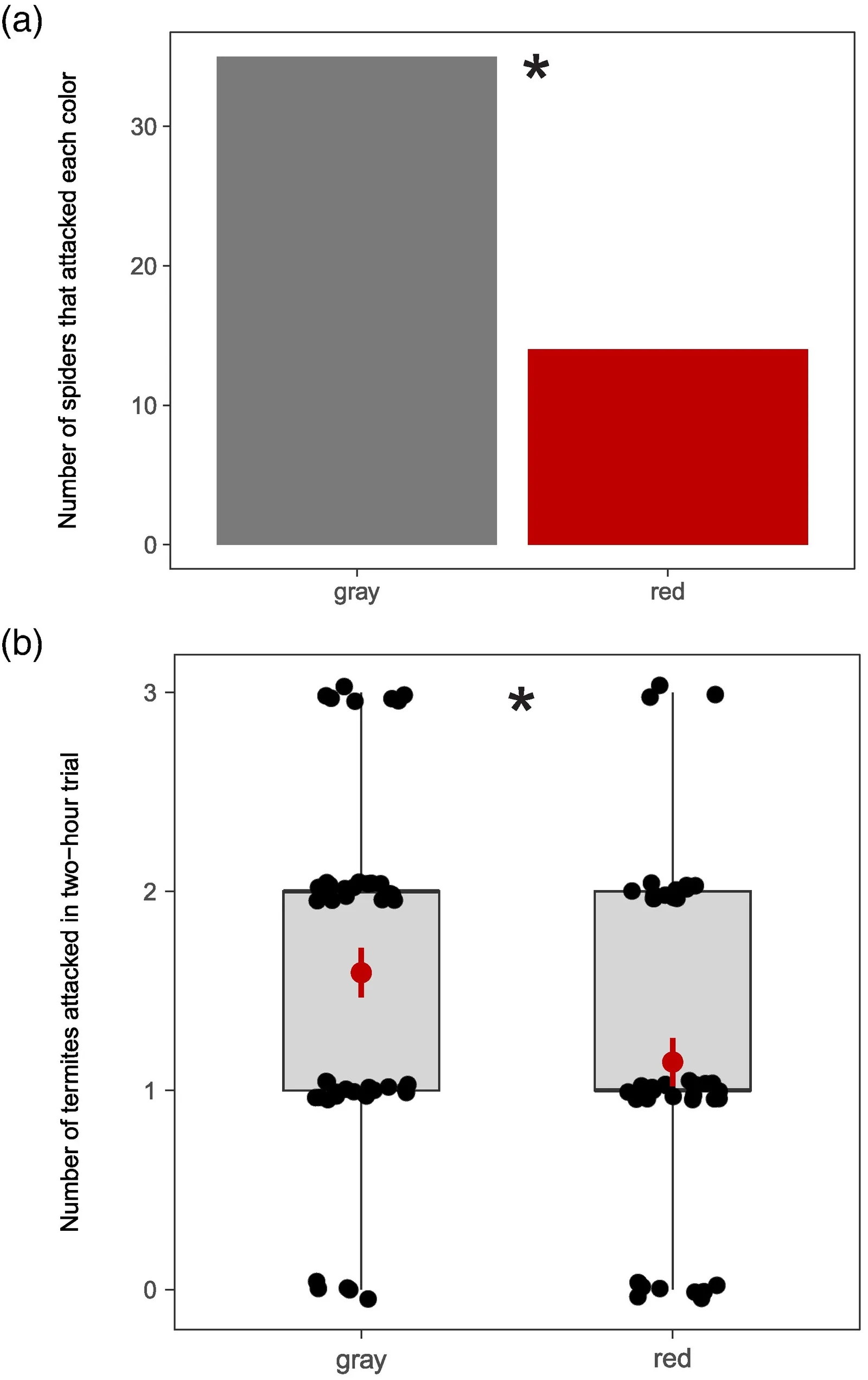
Results of Phase 1 foraging tests to assess whether spiders (*H. pyrrithrix*) learned the association between red color and unpalatability. As expected, after their training (a) significantly fewer spiders directed their first attacks toward the red termites compared with the gray (b) spiders attacked the red termites fewer times compared with the gray over the entire 2-h trial. Box plots show the median and interquartile range; the vertical red line shows the mean (as a red dot) ± SE. Raw data points are shown in gray.

#### Exploratory tests

We found no evidence that the spiders were using brightness cues (independent of color) to make their choices. When the brightness level of each termite (1, 2, or 3) was treated as a categorical predictor variable, we found no differences in the brightness category for the termites they first attacked (20 chose level 1, 12 chose level 2, 17 chose level 3; likelihood ratio *X*^2^ = 2.06, *P* = 0.357). Similarly, when the brightness level (1, 2, or 3) was treated as a continuous variable, there was again no bias detected toward brighter or darker colors (2-tailed *t*-test to ask if the mean differs from 2, *t* = −0.489, *P* = 0.627, mean = 1.94 [upper CI = 2.19, lower CI = 1.68]).

When qualitatively assessing the patterns of pairwise comparisons between all possible R–G combinations (at different brightness levels), we found that in 8 out of 9 comparisons, as expected, the gray was more likely to be attacked first compared with the red, and these differences were statistically significant in 4 of these 8 cases (Table S5). Most notably, when we compared R1 vs. G3 (where we know that R1 must be either brighter than or similarly bright to G3, regardless of which photoreceptors mediate brightness perception; see Fig. 1c), G3 was more likely to be attacked first, although this relationship was not statistically significant (G3 = 15, R1 = 9, likelihood ratio *X*^2^ = 1.52, *P* = 0.218, Table S5). When we compared R3 vs. G1 (where we know that G1 must be either brighter than or similarly bright to R3, regardless of which photoreceptors mediate brightness perception; see Fig. 1c), we found that G1 was more likely to be attacked first (G1 = 11, R3 = 2, likelihood ratio *X*^2^ = 6.86, *P* = 0.00088, Table S5).

### Phase 2: assessing whether the ability to use color vision improves foraging success

#### Confirmatory tests

Five females did not participate in Phase 2 (ie they did not attack either of the termites during their allotted testing time; see Materials and methods), and 1 female was excluded from the Phase 2 data analysis due to an error in the key to her DB-treated vs. untreated termite arrangement which caused the blind observer to be unable to collect accurate data for her test trial. The following results are those from the remaining 43 spiders. As expected, when spiders had access to color cues (ie those in the color-present group), they were less likely to direct their first attack toward a DB-treated termite (1-tailed 2 sample test for proportions, *P* = 0.041, Fig. 3a). Also as expected, spiders in the color-present group ate more palatable (untreated) termites over the course of the 2-h trial than those in the color-absent group (1-tailed Wilcoxon rank sum test, *Z* = 1.96, *P* = 0.025, Fig. 3b). Spiders in the color-absent group tended to have more total wasted attacks during the 2-h trials compared with those which had access to color cues but this relationship did not meet the threshold for statistical significance (1-tailed Wilcoxon rank sum test, Z = −1.28, *P* = 0.0995).

**Figure 3:**
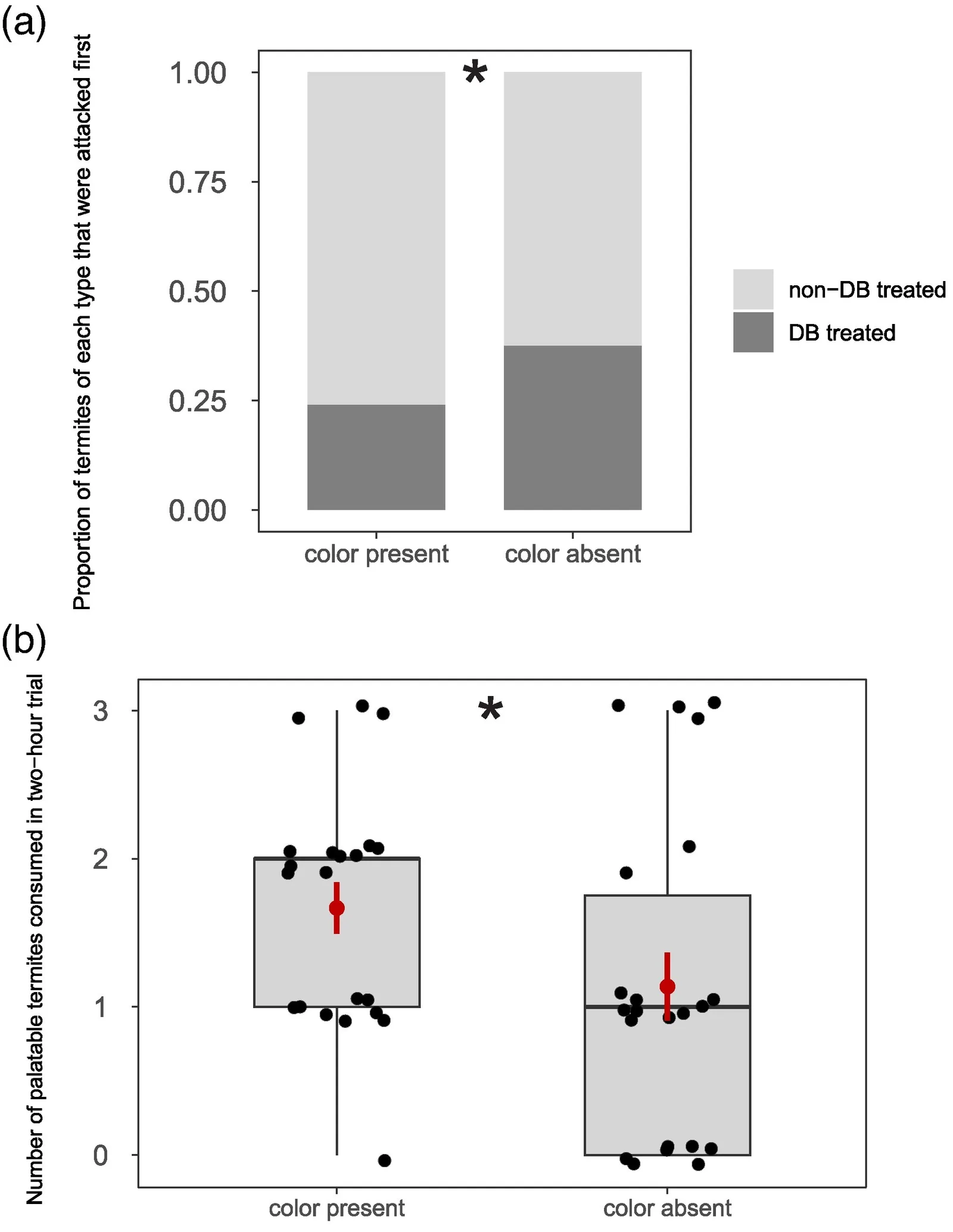
Results of Phase 2 foraging tests to assess whether spiders (*H. pyrrithrix*) that had access to color cues (ie in the color-present treatment group) had higher foraging success. As expected, (a) spiders in the color-present group were better able to avoid the unpalatable (DB-treated) termites on their first attack and (b) spiders in the color-present group consumed more palatable termites in the 2-h trial. Box plots show the median and interquartile range; the vertical red line shows the mean (as a red dot) ± SE. Raw data points are shown in gray.

### Phase 3: assessing whether the ability to use color vision improves fitness

#### Confirmatory tests

As expected, spiders in the color-present group laid their first egg sac faster (in fewer days) than the color-absent group (1-tailed Wilcoxon rank sum test, *Z* = −1.98, *P* = 0.0237, Fig. 4a). Contrary to expectation, spiders in the color-present group did not produce more egg sacs (1-tailed Wilcoxon rank sum test, *Z* = 0.234, *P* = 0.593, total egg sacs ranged from 0 to 2), more total eggs (1-tailed Wilcoxon rank sum test, *Z* = 0.624, *P* = 0.734, total eggs ranged from 0 to 39), nor more hatchlings (1-tailed Wilcoxon rank sum test, *Z* = 1.19, *P* = 0.883, total number of hatchlings ranged from 0 to 29) by the end of the experiment compared with those in the color-absent group.

**Figure 4:**
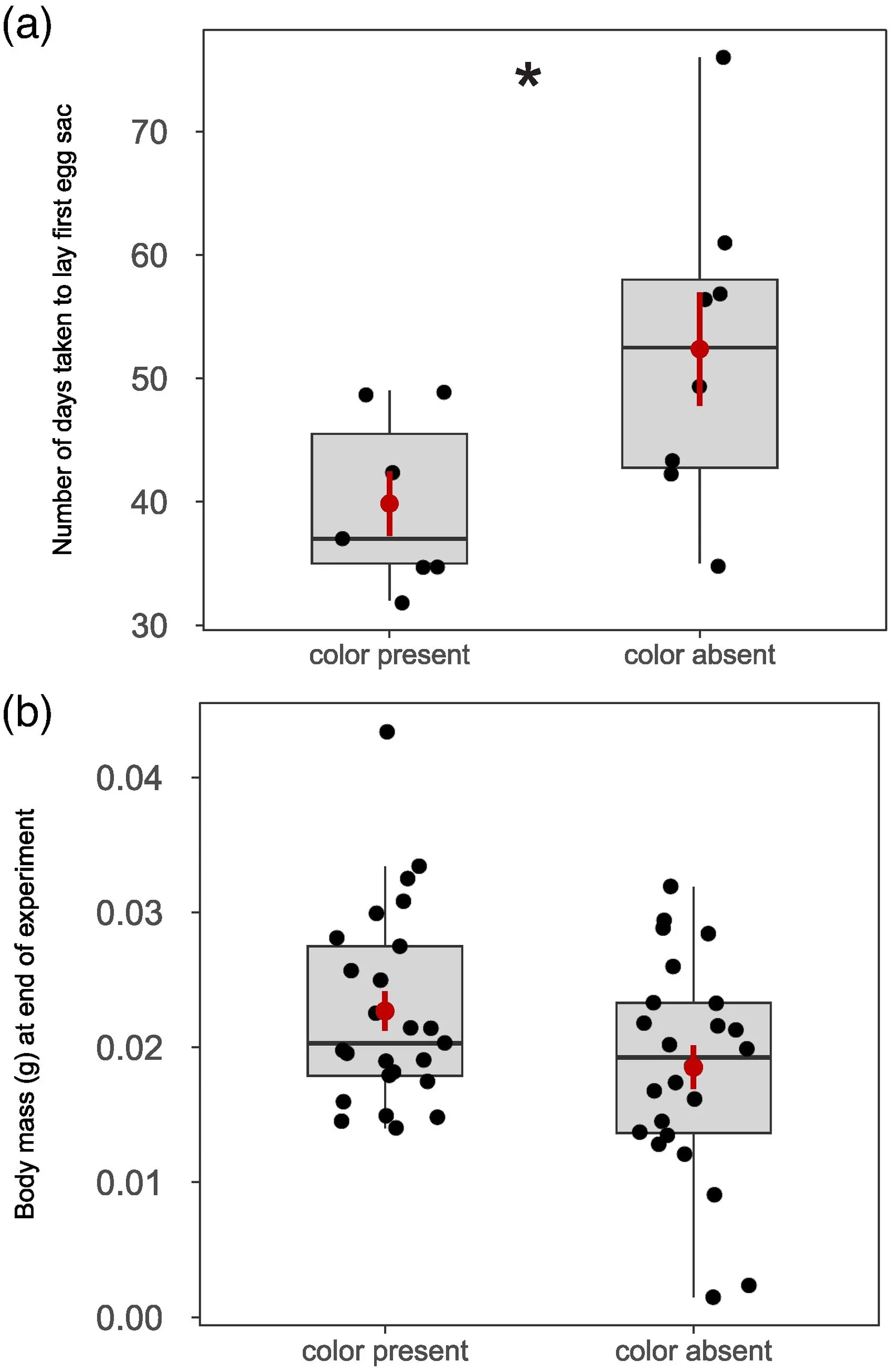
Results of Phase 3 tests to assess whether spiders (*H. pyrrithrix*) that had access to color cues (ie in the color-present treatment group) had higher measures of fitness after the 60-d experimental pipeline. As expected, (a) spiders in the color-present group laid eggs sooner (ie in fewer days) and (b) tended to be heavier at the end of the experiment compared with those in the color-absent group. Box plots show the median and interquartile range; the vertical red line shows the mean (as a red dot) ± SE. Raw data points are shown in gray.

#### Exploratory tests

Spiders in the color-present group tended to be heavier at the end of the experiment than spiders in the color-absent group, but this relationship did not meet the threshold for statistical significance (2-tailed *t*-test, t = 1.93, *P* = 0.0595, Fig. 4b).

## Discussion

Here, we experimentally manipulated access to color cues in a visual predator and found evidence that color vision (ie, the ability to use red color cues, independent of brightness) improved foraging success. After being successfully trained to avoid red prey, spiders with access to red color cues more accurately directed attacks toward palatable termites and away from unpalatable ones, consumed more palatable termite prey, and tended to waste fewer attacks (toward unpalatable prey) over a 2-h foraging task. We found no evidence that brightness affected which termites were attacked, and this is not surprising given that our experiments were designed to make redness, rather than brightness, the most reliable indicator of unpalatability. After 2 mo in our experimental pipeline, spiders that consistently had access to red color cues during feeding produced egg sacs faster and had a trend toward higher body mass, suggesting that they may have been more likely to be gravid again. However, the 2 treatment groups did not differ in the total number of egg sacs, eggs, or hatchlings produced by the end of the experiment. To our knowledge, this is one of the very few empirical studies that have directly tested the fitness effects of color vision in any animal.

In Phase 1 of our experiment, we successfully confirmed that, after their training, the spiders showed an aversion to redness in termites, independent of brightness. This work builds on previous studies of color use and color learning in *H*. *pyrrithrix*. Previous work had already shown that these spiders respond to and learn red color cues when foraging, but these studies did not vary brightness cues independently from color; as such, prior results had to be interpreted more cautiously, because we could not always be confident that the spiders were responding to redness, independent of brightness or contrast (Taylor et al. 2014, 2016; Vickers and Taylor 2018, 2020; Powell et al. 2019). Here, by varying both redness and brightness cues independently (and analyzing the effects of both), we can conclude more confidently that the spiders were making their choices in response to redness and not brightness. Our approach and rationale here were similar to that of the “gray-card” experiments used by von Frisch to establish color vision in honeybees in the early 1900s, where bees were asked to distinguish colored cards from various shades of gray; such approaches are still regarded as solid evidence of color vision today (reviewed in Kelber et al. 2003). In addition, we are fortunate to now have species-specific visual models for *H. pyrrithrix* that take into account measurements of photoreceptor sensitivities and allow us to model what colors our spiders can see and discriminate (Zurek et al. 2015; Maia et al. 2019; see Materials and methods). Yet, further understanding of the complex physiology and neural processes underlying spider vision is required to be able to confidently use visual models to quantify brightness perception and create experiments with perfectly brightness-matched stimuli that differ only in color. As our visual modeling showed, iterations of the models that set different photoreceptors to mediate brightness produce vastly different results (see Materials and methods, Tables S2, S3). For this reason, behavioral experiments that vary both redness and brightness independently and incorporate insight from the visual models cautiously as we have done here are particularly informative. It is also important to note that the aversions to red that we documented at the end of Phase 1 may have resulted from some combination of innate biases, experiences learned in the field prior to collection, and experiences learned in the lab; our goal here was not to disentangle these effects, but simply to ensure that the spiders had this aversion to red so that they could move on to Phases 2 and 3 of our study.

In Phase 2 of our experiment, we found clear evidence that the spiders' ability to use color cues increased their foraging success, at least when performing relatively simple tasks in a controlled environment. We designed our experiment to present an ecologically relevant foraging challenge to these spiders: we trained spiders by giving them a consistent signal (redness) that was always paired with unpalatability. Lack of redness (gray) was always paired with palatability. Because unpalatability was induced using DB, spiders could not detect it without making contact (see additional details in Materials and methods; see also Winsor et al. 2020), meaning that redness was the only reliable cue for avoiding unpalatable prey before attack. This experimental design is modeled after what the spiders might experience in nature, as many small insects advertise chemical defenses with red warning signals (Ruxton et al. 2004). Training under simple, consistent rules is known to facilitate learning (Shettleworth 2010), but natural environments are far more complex. In nature, predators have to navigate diverse suites of prey with different colors and modes of defense, and different and nuanced “rules” about how color might be tied to defense. They also may have to attend to other cues at the same time, such as those needed to find prey (when they are not presented neatly in a petri dish) and to avoid predators. Having now established that color cues can improve foraging in a simplified context, future work can test if and how these benefits play out in more complex and variable environments.

In Phase 3 of our experiment, we found some evidence for longer-term fitness benefits of color vision. The finding that spiders with access to color cues laid their first egg sacs earlier than those that did not, and tended to be heavier at the end of the experiment, suggests that they would likely have laid eggs again if the experiment had not ended at day 60. It is important to note that in previous studies with *H*. *pyrrithrix*, fertilized females given unlimited access to food (crickets) consistently laid eggs within 14 d of mating (Taylor and McGraw 2013). In the present study, spiders took substantially longer to lay their first egg sac (39.9 d in the color-present group and 52.4 d in the color-absent group), suggesting that our experiment captured meaningful variation in food limitation. This is likely because the spiders in our experiment had to carefully navigate palatable and unpalatable prey options as they might in the field, rather than simply consuming unlimited crickets. Laying eggs sooner may translate to several possible benefits in the field. In the Arizona population where these spiders were collected, females most often lay eggs from spring until late summer, and their offspring typically overwinter as juveniles (Taylor et al. 2011). As such, females that lay eggs earlier in the season may have a larger reproductive window to lay additional eggs before conditions get too cold at the end of the season. Moreover, spiderlings that hatch earlier may reach larger sizes ahead of winter, giving them a survival or reproductive advantage going into the next breeding season. Additionally, *H*. *pyrrithrix* is highly cannibalistic (Taylor 2012; Taylor et al. 2017) and spiderlings that are bigger earlier in the season may experience lower predation risk by conspecifics (Taylor 2012), as well as by congenerics (Sung et al. 2025) or other predators (Taylor et al. 2019). Laying eggs sooner may also increase the chances that females will reproduce at all; when predation risk is high, many females are likely killed by mud dauber wasps (Taylor et al. 2019) or parasitized by ichneumonid wasps (Takasuka and Broad 2024) or mantisflies (Redborg 1998) before they get a chance to reproduce.

One question that remains from our study is why the clear increase in foraging success that we saw in Phase 2 did not result in many of the other longer-term fitness measures that we expected (ie an increase in the total number of egg sacs, eggs, and hatchlings produced). There are several ways to think about these data. First, because our study design manipulated access to color cues in adult females that had already mated (see our detailed rationale in Materials and methods), it did not capture how the ability to use color cues during juvenile/subadult foraging might impact lipid availability for oogenesis at adulthood (Kleinteich et al. 2015). In at least some spiders, adult feeding can result in total reproductive recovery over a long enough time period (Dong et al. 2025), but this has not been tested explicitly in jumping spiders, which have a shorter reproductive window. In addition, it is possible that our 60-d experimental window beyond mating may have been too short to fully capture longer-term fitness effects. The fact that we did see some expected fitness effects (ie faster time to egg laying and a trend toward being heavier at the end of the experiment) suggests that if we had extended our experiment, the compounded effect of foraging success would have had more time to translate into more eggs and spiderlings. Ideally, future experiments should track lifetime reproductive success in conditions that mimic those in the field. However, this is incredibly time-consuming. Even for the 60-d pipeline used in the present study, providing 6 termites 3 times per week for 49 female spiders meant that nearly 1,000 termites had to be acquired and delicately painted each week, transferred to individual Petri dishes fitted with custom-cut filter paper, and then placed into the spider houses. Extending such an effort to encompass lifetime reproductive success is not trivial. However, we still argue that these experiments are critically needed, worthwhile endeavors, and clearly, short-lived arthropods are an excellent place to start.

An alternative possibility for why we did not see the expected differences in reproductive output between our treatment groups may be that the spiders without access to color cues compensated and learned how to avoid the DB-treated termites in other ways that did not require attending to color cues. Many species of jumping spiders are known to use trial-and-error learning for foraging tasks (eg Jackson 2002; Jackson and Nelson 2011), and so it would not be surprising if the spiders used such a strategy here. Our experiment was designed such that redness was the only reliable indicator of unpalatability that spiders could assess before attacking. That meant that spiders in the “color-absent” treatment group should have had no way to tell whether a termite was palatable or unpalatable. To compensate for this, they could have adopted a strategy where they cautiously “tasted” the termite before launching a full-on attack; we have observed such cautious “taste/touch and reject” behaviors in the field and lab in this species, most notably when these spiders are navigating novel or particularly risky prey (pers. obs.). Notably, our Phase 2 results were recorded early in our experimental pipeline; future iterations of this experiment should give them a similar foraging test at day 60 to assess whether the 2 treatment groups diverge in their strategies as the experiment progresses. We might expect that the most effective strategy will depend on the information that spiders have access to; specifically, in the “color-present” treatment group, spiders may continue to use redness to navigate their choices, while spiders in the “color-absent” group might adopt a taste-before-attacking strategy. There is precedent in the broader literature for expecting that animals may develop different foraging strategies depending on how well their color vision works; in the monkey studies reviewed in the introduction, dichromatic individuals were thought to compensate for their limited color vision by using olfaction to find ripe fruits (Melin et al. 2009).

In conclusion, here we show that color vision (ie the ability to use redness as a cue to avoid unpalatable prey) improves foraging success and accelerates reproduction in a visually guided generalist predator. However, this might not be the only way that color vision improves foraging in this species. Current work is exploring the idea that color vision may also help these spiders find palatable, colorful prey on colorful backgrounds (eg a brown cricket on a green leaf, or a green grasshopper on a reddish leaf). Moreover, any discussion of color vision in *H. pyrrithrix* must also be placed within the broader context of sexual selection, given the striking red coloration of male faces that appear to be important in courtship interactions (eg Taylor and McGraw 2013; Echeverri et al. 2017). Indeed, the selective pressures that shape color vision likely span many contexts beyond foraging, such as mating, predator avoidance, navigation, etc.

Looking beyond *H. pyrrithrix*, can these findings help explain why color vision has evolved independently multiple times across the jumping spider phylogeny? Might other species of jumping spiders that have independently evolved color vision gain the same benefits as *H. pyrrithrix*? Perhaps, but it is important to acknowledge the extraordinary diversity in natural history across the jumping spider phylogeny. So, we cannot assume that color vision benefits all species in similar ways, even if they are all voracious visual predators. Spiders in the genus *Habronattus* appear to be broad generalists, meaning that they forage on a wide variety of prey and may therefore be expected to gain benefits from the ability to learn about and avoid red aposematic prey, as we have shown here. However, color vision has evolved independently in other jumping spider clades (see Outomuro et al. 2019), including *Stenaelurillus*, which are predominantly termite specialists (Pekár et al. 2020), and thus may be expected to have less reason to attend to redness in prey. There is also some behavioral evidence of color vision in *Evarcha culicivora*, a mosquito specialist that appears to use red-colored cues to find blood-filled mosquitoes (Taylor et al. 2022). With nearly 7,000 species and several independent origins of color vision, the jumping spiders are a fascinating group to help us explore the fitness benefits of color vision and, ultimately, to understand why color vision in its various forms evolves in some groups of animals but not others.

## Supplementary Material

arag110_Supplementary_Data

## Data Availability

Analyses reported in this article can be reproduced using the data provided by Powell et al. (2026).

## References

[arag110-B1] Andersson M . 1994. Sexual selection. Princeton Univ Press. 10.1515/9780691207278.

[arag110-B2] ASAB Ethical Committee/ABS Animal Care Committee . 2024. Guidelines for the ethical treatment of nonhuman animals in behavioural research and teaching. Anim Behav. 207:i–xi. 10.1016/S0003-3472(23)00317-2.

[arag110-B3] Bradley BJ, Lawler RR. 2011. Linking genotypes, phenotypes, and fitness in wild primate populations. Evol Anthropol. 20:104–119. 10.1002/evan.20306. 22034168

[arag110-B4] Brower LP . 1969. Ecological chemistry. Sci Am. 220:22–29. 10.1038/scientificamerican0269-22. 5767170

[arag110-B5] Caine NG, Osorio D, Mundy NI. 2010. A foraging advantage for dichromatic marmosets (*Callithrix geoffroyi*) at low light intensity. Biol Lett. 6:36–38. 10.1098/rsbl.2009.0591. PMC2817255 19740895

[arag110-B6] Carducci JP, Jakob EM. 2000. Rearing environment affects behaviour of jumping spiders. Anim Behav. 59:39–46. 10.1006/anbe.1999.1282. 10640365

[arag110-B7] Caves EM et al 2025. Emerging frontiers in visual ecology. J Exp Biol. 228:jeb250537. 10.1242/jeb.250537. 40693401

[arag110-B8] Cross FR, Jackson RR, Taylor LA. 2020. Influence of seeing a red face during the male–male encounters of mosquito-specialist spiders. Learn Behav. 48:104–112. 10.3758/s13420-020-00411-y. 31975326

[arag110-B9] Dong Y, Gols R, Harvey JA. 2025. Food availability influences adult body mass variability and reproductive traits in a spider. Oecologia. 207:179. 10.1007/s00442-025-05818-w. 41152547

[arag110-B10] Echeverri SA, Morehouse NI, Zurek DB. 2017. Control of signaling alignment during the dynamic courtship display of a jumping spider. Behav Ecol. 28:1445–1453. 10.1093/beheco/arx107.

[arag110-B11] Fedigan LM, Melin AD, Addicott J, Kawamura S. 2014. The heterozygote superiority hypothesis for polymorphic color vision is not supported by long-term fitness data from wild neotropical monkeys. PLoS One. 9:e84872. 10.1371/journal.pone.0084872. PMC3880319 24404195

[arag110-B12] Flamarique IN . 2016. Diminished foraging performance of a mutant zebrafish with reduced population of ultraviolet cones. Proc Biol Sci. 283:20160058. 10.1098/rspb.2016.0058. PMC4810871 26936243

[arag110-B13] Gamberale-Stille G, Tullberg BS. 1999. Experienced chicks show biased avoidance of stronger signals: an experiment with natural colour variation in live aposematic prey. Evol Ecol. 13:579–589. 10.1023/A:1006741626575.

[arag110-B14] Halpin CG et al 2020. Pattern contrast influences wariness in naïve predators towards aposematic patterns. Sci Rep. 10:9246. 10.1038/s41598-020-65754-y. PMC7280217 32514003

[arag110-B15] Hill GE . 2006. Female choice for ornamental coloration. In: Hill GE, McGraw KJ, editors. Bird coloration: function and evolution, Vol. 2. Harvard Univ Press. p. 137–200. 10.2307/j.ctv22jnr8k.

[arag110-B16] Hiwatashi T et al 2010. An explicit signature of balancing selection for color-vision variation in New World monkeys. Mol Biol Evol. 27:453–464. 10.1093/molbev/msp262. 19861643

[arag110-B17] Powell E, Stalcup K, Taylor L. 2026. Data from: Color vision improves foraging success and accelerates reproduction in a jumping spider. Behavioral Ecology. 10.5061/dryad.15dv41pbf.

[arag110-B18] Humbel E, Kimball R, Taylor LA. 2023. The role of high-contrast male facial stripes in mitigating female aggression in the jumping spider *Plexippus paykulli*. Anim Behav. 206:13–27. 10.1016/j.anbehav.2023.08.006.

[arag110-B19] Ihle M, Winney IS, Krystalli A, Croucher M. 2017. Striving for transparent and credible research: practical guidelines for behavioral ecologists. Behav Ecol. 28:348–354. 10.1093/beheco/arx003. PMC5873838 29622916

[arag110-B20] Jackson R . 2002. Trial-and-error derivation of aggressive-mimicry signals by *Brettus* and *Cyrba*, spartaeine jumping spiders (Araneae: Salticidae) from Israel, Kenya, and Sri Lanka. N. Z. J. Zool. 29:95–117. 10.1080/03014223.2002.9518294.

[arag110-B21] Jackson RR, Nelson XJ. 2011. Reliance on trial and error signal derivation by *Portia africana*, an araneophagic jumping spider from East Africa. J Ethol. 29:301–307. 10.1007/s10164-010-0258-5.

[arag110-B22] Kelber A, Osorio D. 2010. From spectral information to animal colour vision: experiments and concepts. Proc Biol Sci. 277:1617–1625. 10.1098/rspb.2009.2118. PMC2871852 20164101

[arag110-B23] Kelber A, Vorobyev M, Osorio D. 2003. Animal colour vision — behavioural tests and physiological concepts. Biol Rev Camb Philos Soc. 78:81–118. 10.1017/S1464793102005985. 12620062

[arag110-B24] Kleinteich A, Wilder SM, Schneider JM. 2015. Contributions of juvenile and adult diet to the lifetime reproductive success and lifespan of a spider. Oikos. 124:130–138. 10.1111/oik.01421.

[arag110-B25] Li J et al 2008. UVB-based mate-choice cues used by females of the jumping spider *Phintella vittata*. Curr Biol. 18:699–703. 10.1016/j.cub.2008.04.020. 18450445

[arag110-B26] Lim ML, Li D. 2004. Courtship and male-male agonistic behaviour of *Cosmophasis umbratica* Simon, an ornate jumping spider (Araneae: Salticidae) from Singapore. Raffles Bull. Zool. 52:435–448.

[arag110-B27] Lunau K, Maier EJ. 1995. Innate colour preferences of flower visitors. J Comp Physiol A. 177:1–19. 10.1007/BF00243394.

[arag110-B28] Maia R, Gruson H, Endler JA, White TE. 2019. Pavo 2: new tools for the spectral and spatial analysis of colour in R. Methods Ecol Evol. 10:1097–1107. 10.1111/2041-210X.13174.

[arag110-B29] Mappes J, Marples N, Endler JA. 2005. The complex business of survival by aposematism. Trends Ecol Evol. 20:598–603. 10.1016/j.tree.2005.07.011. 16701442

[arag110-B30] Marples NM, Brakefield PM, Cowie RJ. 1989. Differences between the 7-spot and 2-spot ladybird beetles (Coccinellidae) in their toxic effects on a bird predator. Ecol Entomol. 14:79–84. 10.1111/j.1365-2311.1989.tb00756.x.

[arag110-B31] Melin AD et al 2009. Fig foraging by dichromatic and trichromatic *Cebus capucinus* in a tropical dry forest. Int J Primatol. 30:753–775. 10.1007/s10764-009-9383-9.

[arag110-B32] Melin AD et al 2017. Trichromacy increases fruit intake rates of wild capuchins (*Cebus capucinus imitator*). Proc Natl Acad Sci U S A. 114:10402–10407. 10.1073/pnas.1705957114. PMC5625910 28894009

[arag110-B33] Melin AD, Fedigan LM, Hiramatsu C, Sendall CL, Kawamura S. 2007. Effects of colour vision phenotype on insect capture by a free-ranging population of white-faced capuchins (*Cebus capucinus*). Anim Behav. 73:205–214. 10.1016/j.anbehav.2006.07.003.

[arag110-B34] Merilaita S, Stevens M. 2011. Crypsis through background matching. In: Stevens M, Merilaita S, editors. Animal camouflage: mechanisms and function. Cambridge University Press. p. 17–33. 10.1017/CBO9780511852053.002.

[arag110-B35] Montgomerie R . 2006. Analyzing colors. In: Hill GE, McGraw KJ, editors. Bird coloration: mechanisms and measurements. Harvard Univ Press. p. 90–147. 10.2307/j.ctv22jnscm.6.

[arag110-B36] Montgomerie R . 2008. CLR, version 1.05. Queen's University.

[arag110-B37] Nosek BA, Ebersole CR, DeHaven AC, Mellor DT. 2018. The preregistration revolution. Proc Natl Acad Sci U S A. 115:2600–2606. 10.1073/pnas.1708274114. PMC5856500 29531091

[arag110-B38] Ogawa Y, Kinoshita M, Stavenga DG, Arikawa K. 2013. Sex-specific retinal pigmentation results in sexually dimorphic long-wavelength-sensitive photoreceptors in the eastern pale clouded yellow butterfly *Colias erate*. J Exp Biol. 216:1916–1923. 10.1242/jeb.083485. 23393285

[arag110-B39] Outomuro D et al 2019. The evolution of colour vision across jumping spiders (conference abstract). Integr Comp Biol. 59:E176.

[arag110-B40] Peaslee AG, Wilson G. 1989. Spectral sensitivity in jumping spiders (Araneae, Salticidae). J Comp Physiol A. 164:359–363. 10.1007/BF00612995. 2709341

[arag110-B41] Pekár S, Dušátková LP, Michálek O, Haddad CR. 2020. Coexistence of two termite-eating specialists (Araneae). Ecol. Entom. 45:1307–1317. 10.1111/een.12914.

[arag110-B42] Pessoa DMA et al 2014. The adaptive value of primate color vision for predator detection. Am J Primatol. 76:721–729. 10.1002/ajp.22264. 24535839

[arag110-B43] Powell EC et al 2019. Prey colour biases in jumping spiders (*Habronattus brunneus*) differ across populations. Ethology. 125:351–361. 10.1111/eth.12859.

[arag110-B44] Raška J, Štys P, Exnerová A. 2018. Perception of olfactory aposematic signals by jumping spiders. Ethology. 124:773–776. 10.1111/eth.12811.

[arag110-B45] R Core Team . 2026. R: a language and environment for statistical computing. R Foundation for Statistical Computing. Available from: https://www.R-project.org. Accessed 2026 Jan 20.

[arag110-B46] Redborg KE . 1998. Biology of the Mantispidae. Annu Rev Entomol. 43:175–194. 10.1146/annurev.ento.43.1.175. 15012388

[arag110-B47] Rettke SP . 2023. Color vision variation, feeding behavior, and reproductive output in a group of wild Ateles belzebuth [Master’s Thesis] University of Texas at Austin.

[arag110-B48] Rivera C, Hedin M, Mason AC, Maddison WP, Elias DO. 2021. Complex courtship in the *Habronattus clypeatus* group (Araneae: Salticidae). J Arachnol. 48:221–232. 10.1636/JoA-S-18-045.

[arag110-B49] Ruxton GD, Neuhäuser M. 2010. When should we use one-tailed hypothesis testing? Methods Ecol Evol. 1:114–117. 10.1111/j.2041-210X.2010.00014.x.

[arag110-B50] Ruxton GD, Sherratt TN, Speed MP. 2004. Avoiding attack: the evolutionary ecology of crypsis, warning signals, and mimicry. Oxford Univ Press. 10.1093/acprof:oso/9780198528609.001.0001.

[arag110-B51] Shettleworth SJ . 2010. Cognition, evolution, and behavior. 2nd edn. Oxford University Press.

[arag110-B52] Simmons LW . 2017. Guidelines for transparency and openness (TOP). Behav Ecol. 28:347–347. 10.1093/beheco/arx019.

[arag110-B53] Somanathan H, Borges RM, Warrant EJ, Kelber A. 2008. Nocturnal bees learn landmark colours in starlight. Curr Biol. 18:R996–R997. 10.1016/j.cub.2008.08.023. 19000807

[arag110-B54] Stevens M, Ruxton GD. 2012. Linking the evolution and form of warning coloration in nature. Proc Biol Sci. 279:417–426. 10.1098/rspb.2011.1932. PMC3234570 22113031

[arag110-B55] Stuckert AM, Summers K. 2023. Investigating signal modalities of aposematism in a poison frog. J Evol Biol. 36:1003–1009. 10.1111/jeb.14111. 36309965

[arag110-B56] Sugiura S, Sato T. 2018. Successful escape of bombardier beetles from predator digestive systems. Biol Lett. 14:20170647. 10.1098/rsbl.2017.0647. PMC5830659 29438051

[arag110-B57] Sung JY, Fogle JA, Morehouse NI. 2025. Spatial overlap and behavioral interactions among four *Habronattus* jumping spider species in a mixed-species assemblage. Ecol Evol. 15:e70871. 10.1002/ece3.70871. PMC11955511 40170805

[arag110-B58] Surridge AK, Osorio D, Mundy NI. 2003. Evolution and selection of trichromatic vision in primates. Trends Ecol Evol. 18:198–205. 10.1016/S0169-5347(03)00012-0.

[arag110-B59] Takasuka K, Broad GR. 2024. A bionomic overview of spider parasitoids and pseudo-parasitoids of the ichneumonid wasp subfamily Pimplinae. Contrib Zool. 93:1–106. 10.1163/18759866-bja10053.

[arag110-B60] Taylor LA . 2012. Color and communication in Habronattus jumping spiders: tests of sexual and ecological selection [Doctoral Dissertation] Arizona State University.

[arag110-B61] Taylor LA, Amin Z, Maier EB, Byrne KJ, Morehouse NI. 2016. Flexible color learning in an invertebrate predator: *Habronattus* jumping spiders can learn to prefer or avoid red during foraging. Behav Ecol. 27:520–529. 10.1093/beheco/arv182.

[arag110-B62] Taylor LA, Clark DL, McGraw KJ. 2011. Condition dependence of male display coloration in a jumping spider (*Habronattus pyrrithrix*). Behav Ecol Sociobiol. 65:1133–1146. 10.1007/s00265-010-1127-5.

[arag110-B63] Taylor LA, Cook C, McGraw KJ. 2019. Variation in activity rates may explain sex-specific dorsal color patterns in *Habronattus* jumping spiders. PLoS One. 14:e0223015. 10.1371/journal.pone.0223015. PMC6795386 31618242

[arag110-B64] Taylor LA, Cross FR, Jackson RR. 2022. Blood-red colour as a prey choice cue for mosquito specialist predators. Anim Behav. 188:85–97. 10.1016/j.anbehav.2022.04.003.

[arag110-B65] Taylor LA, Maier EB, Byrne KJ, Amin Z, Morehouse NI. 2014. Colour use by tiny predators: jumping spiders show colour biases during foraging. Anim Behav. 90:149–157. 10.1016/j.anbehav.2014.01.025.

[arag110-B66] Taylor LA, McGraw KJ. 2013. Male ornamental coloration improves courtship success in a jumping spider, but only in the sun. Behav Ecol. 24:955–967. 10.1093/beheco/art011.

[arag110-B67] Taylor LA, Powell EC, Mcgraw KJ. 2017. Frequent misdirected courtship in a natural community of colorful *Habronattus* jumping spiders. PLoS One. 12:e0173156. 10.1371/journal.pone.0173156. PMC5381776 28379968

[arag110-B68] van der Kooi CJ, Dyer AG, Kevan PG, Lunau K. 2019. Functional significance of the optical properties of flowers for visual signalling. Ann Bot. 123:263–276. 10.1093/aob/mcy119. PMC6344213 29982325

[arag110-B69] van der Kooi CJ, Stavenga DG, Arikawa K, Belušič G, Kelber A. 2021. Evolution of insect color vision: from spectral sensitivity to visual ecology. Annu Rev Entomol. 66:435–461. 10.1146/annurev-ento-061720-071644. 32966103

[arag110-B70] Vickers ME, Taylor LA. 2018. Odor alters color preference in a foraging jumping spider. Beh. Ecol. 29:833–839. 10.1093/beheco/ary068. PMC6041943 30018487

[arag110-B71] Vickers ME, Taylor LA. 2020. Hemipteran defensive odors trigger predictable color biases in jumping spider predators. Sci Rep. 10:21898. 10.1038/s41598-020-78952-5. PMC7736339 33318578

[arag110-B72] von Schantz TV, Bensch S, Grahn M, Hasselquist D, Wittzell H. 1999. Good genes, oxidative stress and condition–dependent sexual signals. Proc Biol Sci. 266:1–12. 10.1098/rspb.1999.0597. PMC1689644 10081154

[arag110-B73] Wickham H . 2016. Ggplot2: elegant graphics for data analysis. Springer-Verlag. Available from: https://ggplot2.tidyverse.org. Accessed 2026 Jan 20.

[arag110-B74] Williams DS, McIntyre P. 1980. The principal eyes of a jumping spider have a telephoto component. Nature. 288:578–580. 10.1038/288578a0.

[arag110-B75] Winsor AM, Ihle M, Taylor LA. 2020. Methods for independently manipulating palatability and color in small insect prey. PLoS One. 15:e0231205. 10.1371/journal.pone.0231205. PMC7138310 32255810

[arag110-B76] World Spider Catalog . 2026. World Spider Catalog. Version 26. Natural History Museum Bern. Available from: http://wsc.nmbe.ch. Accessed 2026 Jan 28. 10.24436/2.

[arag110-B77] Yan L et al 2025. Latent preference for red ornamentation drives interspecific mating in nascent jumping spider species (*Habronattus americanus* group, F. Salticidae). Proc Biol Sci. 292:20242754. 10.1098/rspb.2024.2754. PMC12364577 40829667

[arag110-B78] Zurek DB et al 2015. Spectral filtering enables trichromatic vision in colorful jumping spiders. Curr Biol. 25:R403–R404. 10.1016/j.cub.2015.03.033. 25989075

