# Supplementary material for: Color vision improves foraging success and accelerates reproduction in a jumping spider
Source: Behav Ecol. 2026 Sep 15;37(6):arag110. doi: 10.1093/beheco/arag110 (PMC13658915; doi:10.1093/beheco/arag110)
Supplement: Supplementary file 1 [file arag110_supplementary_data.pdf]

Supplementary material (Tables S1–S5)

Powell EC, Stalcup K, Taylor LA. 2026. Color vision improves foraging success and accelerates reproduction in a jumping spider. Behavioral Ecology.

Table S1. Results of t-tests comparing the mean brightness (% reflectance) values between each paint pair. Asterisks indicate a significant difference between pairs. Our goal in creating these mixtures was to ensure that our red and gray paint pairs were statistically similar in mean brightness (R1=G1, R2=G2, R3=G3) while the different brightness levels were statistically different from one another (R1>R2>R3, G1>G2>G3). Note that these data are independent of the visual system of the viewer.

	R1	R2	R3	G1	G2	G3
**R1**						
**R2**	t_{54}= -79.42, p=0.0001*					
**R3**	t_{54}= -119.54, p=0.0001*	t_{54}= -40.12, p=0.0001*				
**G1**	t_{54}= -1.56, p=0.125	t_{54}= -80.97, p=0.0001*	t_{54}= -121.10, p=0.0001*			
**G2**	t_{54}= 81.07, p=0.0001*	t_{54}= 1.66, p=0.103	t_{54}= -38.46, p=0.0001*	t_{54}= -82.63, p=0.0001*		
**G3**	t_{54}= 119.39, p=0.0001*	t_{54}= 39.97, p=0.0001*	t_{54}= -0.15, p=0.883	t_{54}= -120.95, p=0.0001*	t_{54}= -38.31, p=0.0001*	

Table S2. Results of *Habronattus pyrrithrix* visual models that compare photoreceptor stimulation (quantum catch) using different model permutations with different photoreceptor classes set to mediate brightness perception (all, long, medium, or short). Note that in models using 'all' or 'long', R1, R2, and R3 are higher than G1, G2, and G3, respectively. However, in models using 'medium' or 'short', the reverse is true, with G1, G2, and G3 being higher than R1, R2, and R3, respectively.

	**all**	**long**	**medium**	**short**
**G1**	9.60	9.00	8.72	6.33
**G2**	9.06	8.44	8.19	5.87
**G3**	8.65	8.01	7.79	5.51
**R1**	9.89	9.66	8.21	6.09
**R2**	9.58	9.40	7.67	5.36
**R3**	9.12	8.90	7.43	5.04

Table S3. Results of visual models showing the JND values (i.e.,'just noticeable differences' or dL values in pavo) in achromatic (brightness) contrast. Note that a value of 1 JND indicates that *Habronattus pyrrithrix* should be able to differentiate those two stimuli based on differences in brightness. The higher the JND value (above 1), the better the spiders should be able to differentiate between the two stimuli. There are two important patterns to note here. First, in the models using 'all' or 'long', red will often (but not always) appear brighter to the spiders than gray (most values fall above 1 JND). In contrast, in models using 'medium' or 'short', gray will often (but not always) appear brighter to the spiders than red (again, most values fall above 1 JND). A second pattern to note is that the variation in brightness within each of our paint color categories (red, gray) was substantial. In all comparisons (regardless of the model permutation, see top six rows of the table), comparisons of the different brightness levels (1, 2, 3) were well above 1 JND meaning that the spiders should have been able to see the differences in brightness within the reds and grays.

	**where red is brighter than gray**		**where gray is brighter than red**	
	**all**	**long**	**medium**	**short**
**G1 vs G2**	10.76	11.00	10.17	6.73
**G1 vs G3**	18.80	19.13	17.81	11.53
**G2 vs G3**	8.01	8.05	7.56	4.61
**R1 vs R2**	6.23	5.13	10.12	9.71
**R1 vs R3**	15.26	15.04	14.58	13.63
**R2 vs R3**	9.01	9.89	4.40	3.59
**G1 vs R1**	5.74	12.91	9.77	3.58
**G1 vs R2**	0.50	7.77	20.00	13.51
**G1 vs R3**	9.51	2.12	24.55	17.62
**G2 vs R1**	16.51	24.00	0.40	3.12
**G2 vs R2**	10.26	18.82	9.72	6.47
**G2 vs R3**	1.25	8.88	14.17	10.24
**G3 vs R1**	24.58	32.25	7.96	7.80
**G3 vs R2**	18.30	27.02	2.14	1.82
**G3 vs R3**	9.25	16.99	6.55	5.44

Table S4. Results of visual models showing the JND values (i.e.,'just noticeable differences' or dS values in pavo) in chromatic (color) contrast. Note that a value of 1 JND indicates that *Habronattus pyrrithrix* should be able to differentiate those two stimuli based on differences in color (in this case, redness). The higher the JND value (above 1) the better the spiders should be able to differentiate between the two stimuli. The important pattern to note here is that regardless of which paint pairs are being compared, the spiders should always be able to easily distinguish the red from gray paint based on differences in chromatic cues (i.e., redness).

	**JND (chromatic)**
**G1 vs R1**	13.89
**G1 vs R2**	17.89
**G1 vs R3**	14.79
**G2 vs R1**	14.47
**G2 vs R2**	18.39
**G2 vs R3**	15.28
**G3 vs R1**	14.81
**G3 vs R2**	18.65
**G3 vs R3**	15.52

Table S5. Pairwise comparisons between all possible R-G combinations indicating the number of trials in which each color was attacked first. R indicates red, G indicates gray, and the numbers 1–3 indicate the brightness level (with 1 being the brightest and 3 being the darkest, see Methods). In 8 of the 9 comparisons, the spider attacked the gray first (as expected). Significant P-values are shown in bold.

	**G1 (n=11)**	**G2 (n=8)**	**G3 (n=15)**
**R1 (n=9)**	G1>R1 (P=0.655)	R1>G2 (P=0.808)	G3>R1 (P=0.218)
**R2 (n=4)**	G1>R2 (P=0.065)	G2>R2 (P=0.244)	G3>R2 **(P=0.0092)**
**R3 (n=2)**	G1>R3 **(P=0.0088)**	G2>R3 **(P=0.0496)**	G3>R3 **(P=0.0008)**
